# Observational study on passive leg raising and the autonomic nervous system

**DOI:** 10.14814/phy2.15537

**Published:** 2022-12-21

**Authors:** Søren Søndergaard

**Affiliations:** ^1^ Department of Intensive Care and Neurointensive Stepdown Unit, Elective Surgery Centre Silkeborg Regional Hospital Silkeborg Denmark

**Keywords:** autonomic nervous system, cardiac output, heart rate variability, passive leg raising, preload responsiveness, stroke volume

## Abstract

In the intensive care and perioperative setting, circulation is often supported by intravenous fluid preceded by prediction of fluid responsiveness during a passive leg raising (PLR) maneuver. An increase in stroke volume (SV) or cardiac output (CO) of 10%–15% indicates that the subject may increase the flow upon volume expansion. However, the semi‐recumbent position as an initial position in PLR likely reduces SV by gravitational displacement of central blood volume (CBV) to lower extremities, thereby accentuating volume responsiveness during leg raising in healthy people. Coincident with gravitational perturbations in hemodynamics, remedial changes occur in the autonomic nervous system (ANS), as expressed in spectral power in heart rate variability (HRV). This study aims to clarify these concomitant changes during PLR. A convenience number of healthy volunteers (*N* = 11) were recruited by advertisement in university departments. The subjects were exposed to the established PLR sequence and the heart rate (HR), mean arterial pressure (MAP), SV, and CO were sampled at 1 Hz, while electrocardiogram was recorded at 1000 Hz. Relative powers reflecting autonomic nervous system activity were assessed from spectral analysis of HRV. In response to PLR, SV increased (12.4% ± 8.7%, *p* < 0.0026), while HR (−7.6% ± 4.7%, *p* < 0.0009) and MAP (−7.6% ± 6.9%, *p* < 0.01) decreased, with no change in CO (4.1% ± 12.8%, ns). The HRV low‐frequency component was reduced (−34%; *p* < 0.0095), while the high‐frequency activity increased (78.5%; *p* < 0.0013), with a 63% decrease in the low/high frequency ratio (*p* < 0.0078). Thus, HRV indicated a reduced sympathetic index (semi‐recumbent 0.808 vs. PLR −0.177 a.u., *p* < 0.001) and an increased parasympathetic index (−0.141 to 0.996 a.u., *p* < 0.0001). Gravitational depletion and expansion of CBV during PLR were associated with a counterregulatory autonomic response. Healthy volunteers appeared volume responsive in terms of SV, but not CO. Responses to PLR are influenced by the ANS, and HRV analysis should be included in the assessment of the PLR test.

## Graphical abstract

Passive leg raising is accompanied by distinct changes in parasympathetic and sympathetic indices from semi‐recumbent position to legs passively raised. This may fundamentally affect the central hemodynamic responses to the test and influence the predictive power of the PLR.

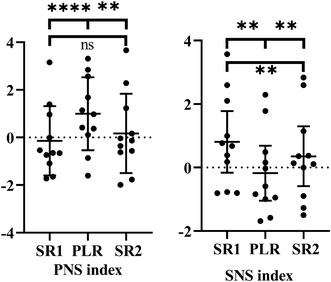

## 1 BACKGROUND

Management of the cardiovascular system in perioperative and intensive care has moved from “one size fits all” in terms of pressure, flow, and fluids to an individualized goal‐directed approach based on—ideally—matching the oxygen delivery (DO_2_) to the oxygen consumption (VO_2_) using fluids, vasoactive, and/or inotropic therapy. To assess the option of volume expansion, an international peer assembly recently emphasized the use of dynamic indices in preload responsiveness, supplanting the indiscriminate use of a fluid bolus which at best elicits a positive response in 50% of patients (Michard & Teboul, 2002). Therefore, pulse pressure variation (PPV), stroke volume variation (SVV), passive leg raising (PLR), and end‐expiratory occlusion test (EEO) are highly recommended as these measures can predict preload responsiveness (De Backer et al., 2019). PLR further carries the endorsement of using an internal fluid bolus avoiding the potentially deleterious effect of external fluid and has been promoted as “five rules and not a drop of fluid” referring to the positioning sequence and the mobilization of blood from the splanchnic area and lower extremities during the procedure (Monnet & Teboul, 2015). However, serious concerns have been raised regarding the physiological groundwork behind the use and interpretation of PPV and SVV (Aneman & Sondergaard, 2016; Bahlmann et al., 2016; Sondergaard, 2013). Likewise, the PLR maneuver deserves scrutiny to clarify its position in the ICU and the perioperative setting. HRV represented by the changes in relative proportions of spectral frequencies in relation to physiological changes (e.g., postural maneuvers) is well recognized and an established tool in research involving ANS (Malik et al., 2019). In this hypothesis‐generating observational pilot study, the hemodynamic and ANS responses to PLR were examined in healthy subjects. The study was pragmatic, used commonly available monitoring equipment, digital signal analysis, and is intended as a precursor to studies in patients with attenuated ANS due to disease, anesthesia, and/or sedation. Sophisticated physiological examinations (e.g., lower body negative pressure, microneurography, electrical impedance tomography, and transcardiac/pulmonary thermodilution) were avoided to obtain general clinical applicability.

### 1.1 Objective

This study aimed to register, describe, and interpret concomitant changes in hemodynamic variables and HRV during PLR.

## 2 METHODS

### 2.1 Test subjects

Eleven healthy subjects (4 F/7 M) were included by advertisements in university departments. Mean ± SD, (range) age 31.5 ± 16.2 years, (22–67), and weight 84.5 ± 12.7 kg, (75–110). Lacking previous data for power calculation, a cohort of 10–15 was deemed reasonable. The subjects were nonsmokers and abstained from alcohol and caffeine‐containing beverages from the day prior to the experiment. To ensure normovolemia, the participants were encouraged to have a normal breakfast, including fluid (e.g., milk products and juice) ad libitum. No fluid was administered during the study. The volunteers were informed in writing and verbally about the study and signed a consent form.

### 2.2 Equipment

The electrocardiogram (ECG) was sampled at 1000 Hz. Finapres (Finapres Medical Systems) continuously and noninvasively measured cardiovascular variables. Based on the Modelflow developed by Peñáz and Wesseling, brachial artery pressure is reconstructed from a reverse transfer function applied to finger arterial blood pressure measured by clamping finger arteries (Bos et al., 1996; Guelen et al., 2003; Peñáz et al., 1976; Wesseling et al., 1993). The equipment calculates SV, MAP, systolic and diastolic blood pressure (SBP, DBP, respectively) adjusted to the phlebostatic axis using the heart reference system (HRS).

### 2.3 Procedure

**FIGURE 1:**
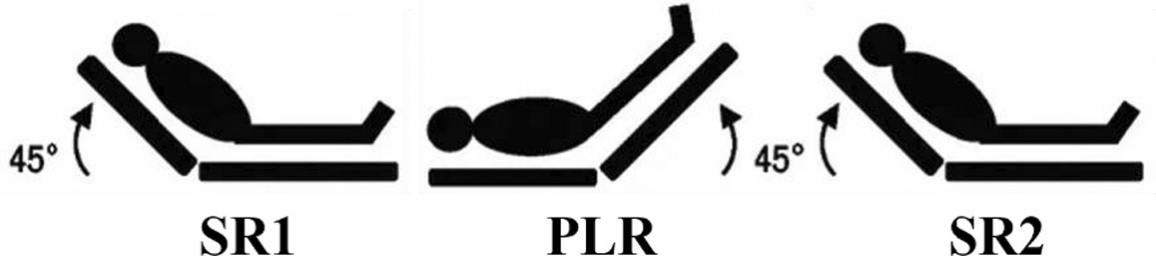
Topological changes during PLR maneuver. PLR, passive leg raising; SR1, initial semi‐recumbent position; SR2, final semi‐recumbent position.

The procedure strictly followed the recommendations of Monnet and Teboul (2015). The subject was placed comfortably on an operating table in a semi‐recumbent position with the legs horizontal (SR1). Baseline values of ECG, SV, CO, HR, MAP, SBP, and DBP were recorded beat‐by‐beat for 5 min. Next, the leg plate was elevated to 45° and the breastplate leveled (PLR). Recordings continued for 10 min before the position was returned to baseline semi‐recumbent for a final 5‐minute recording of said variables (SR2) (see Figure 1).

### 2.4 Analyses

#### 2.4.1 Hemodynamic variables

Representative 5‐minute sequences (250–300 samples) were selected from the Finometer recordings (HR, SBP/DBP, MAP, SV, and CO) and ECG. Sequences were visually assessed as stable and free of artifacts.

#### 2.4.2 Heart rate variability

**FIGURE 2:**
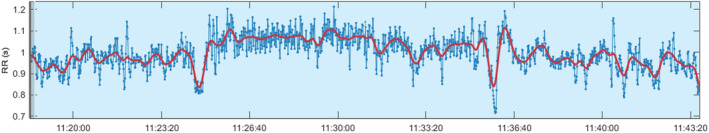
Example of recording of RR intervals during SR1, PLR, and SR2. The two nadirs at approximately 11.24 and 11.35 mark the transition between positions

HRV is a description of the minute variations in the RR interval of the ECG. The spectral analysis dissects the vector of RR intervals as a composite of sinusoidal functions. The intervals are subjected to a Fourier transformation from which primarily the frequencies, phase angle, power, and amplitudes are obtained. In this pilot study, ECG data were exported in the European Data Format (EDF) to Kubios Premium 3.0.1 (Kubios Oy, www.kubios.com) (Figure 2), and the following spectral variables were extracted and reported from automatically corrected 5‐min samples of ECG during SR1, PLR, and SR2: high (HF, 0.15–0.4 Hz) and low (LF, 0.04–0.15 Hz) frequency power of the RR interval and the composite indices of parasympathetic (PNS) and sympathetic (SNS) activity (Tarvainen et al., 2021). PNS increases HRV and is considered a major contributor to the HF component. Conversely, the LF component of HRV is conventionally considered to include both SNS and PNS influence with SNS activity likely being the dominant (Iwase et al., 2017; Malik, 1996; Stick, 1996), accepting that HRV may be influenced by HR itself. The LF/HF ratio accentuates ANS‐engendered changes.

### 2.5 Statistics

All subjects provided analyzable data. After inspection and robust regression and outlier removal (ROUT; Motulsky & Brown, 2006), the normal distribution of the data was assessed using the Kolmogorov–Smirnov test. The differences between SR1 and PLR were tested using paired *t*‐tests for HR, MAP, SV, and CO. Further analyses included one‐way paired Gaussian analysis of variance (ANOVA) of mean values, as warranted by the normality of data (Sullivan et al., 2016). Statistical significance was defined as *p* < 0.05 (GraphPad Prism ver. 8.4 and 9.4.1). In view of the multiple significant results, post hoc power calculation was waived after consultation with in‐hospital statistical expertize (Zhang et al., 2019).

## 3 RESULTS

### 3.1 Hemodynamic variables

**FIGURE 3:**
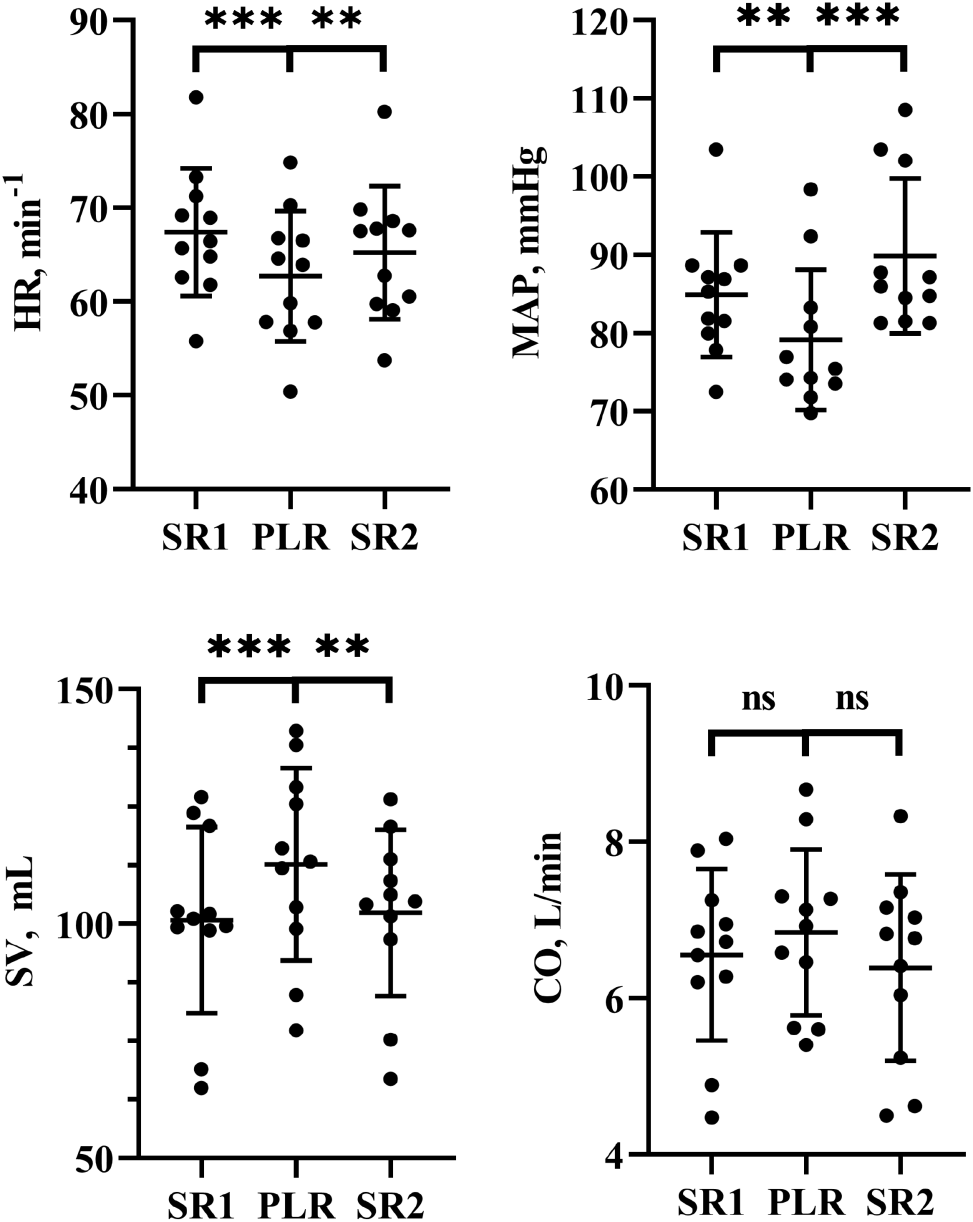
Mean and SD of individual measurements of MAP, HR, SV, and CO during the three phases. Only the changes in CO were insignificant. Ns: *p* > 0.05; **p ≤ 0.01; ***p ≤ 0.001

All variables were normally distributed. Data are presented as the mean ± SD. According to a Δ10%‐criterion of preload responsiveness, 9/11 were responsive in terms of SV which increased by 12.2% ± 8.7% (*p* = 0.0026) during PLR, while there was no significant change in CO (4.1% ± 12.8%), 3/11 were responsive in terms of CO. Conversely, PLR reduced HR (−7.6% ± 4.7%, *p* = 0.0009) and MAP (−7% ± 6.9%, *p* < 0.01). All variables returned to baseline when the subjects were placed in the semi‐recumbent position, SR2 (Figure 3).

### 3.2 Heart rate variables

**FIGURE 4:**
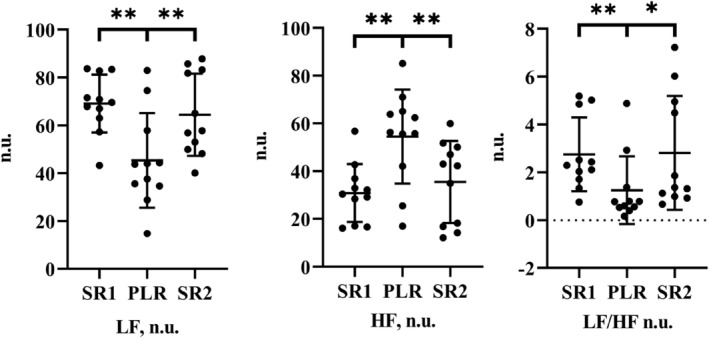
LF, HF, and LF/HF in normalized units during SR1, PLR, and SR2. Differences between positions SR1/2 and PLR are significant. *p ≤ 0.05; **p ≤ 0.01

**FIGURE 5:**
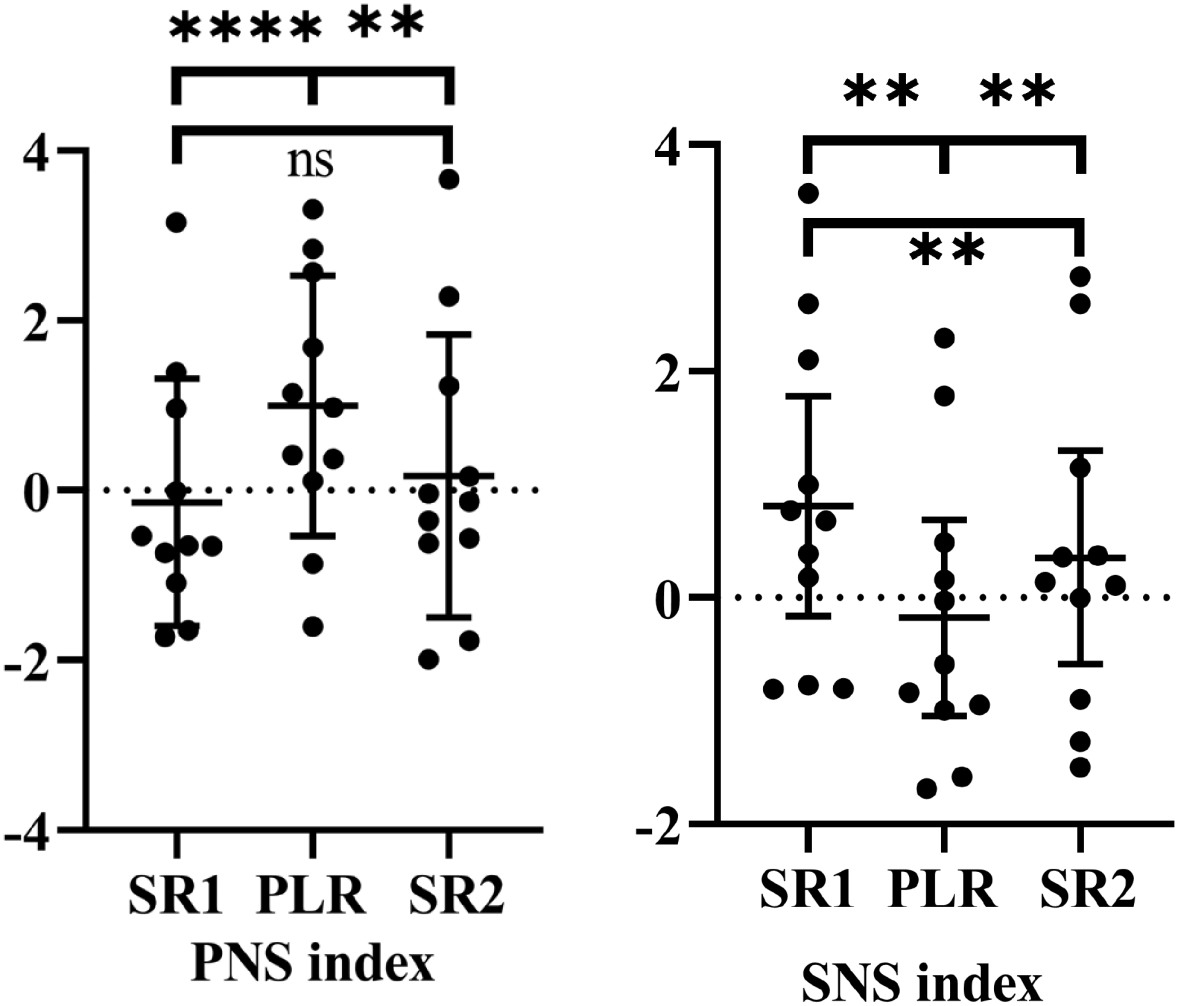
Individual PNS and SNS indices during the three phases. A significant increase was seen in PNS during PLR concomitant with a decrease in SNS. Ns: *p* > 0.05; **p ≤ 0.01; ****p ≤ 0.0001

During PLR, LF power decreased, whereas HF increased; that is, the LF/HF ratio decreased (Figure 4).The PNS index increased from SR1 to PLR (−0.141 to 0.996 a.u.; *p* < 0.0001), while the SNS index decreased (from 0.808 to −0.177 a.u.; *p* < 0.001; Figure 5). In addition, all HRV variables returned to baseline when subjects returned to SR2. The changes in the low and high frequencies in normalized units, as well as the LF/HF ratio, indicated a relative increase in parasympathetic and decrease (withdrawal) in sympathetic power during passive leg raising.

## 4 DISCUSSION

### 4.1 Results

The relationship between PLR and ANS was studied in 11 normovolemic subjects who underwent the standardized PLR test. Hemodynamic variables changed in response to PLR by increasing SV and decreasing MAP and HR, thereby maintaining CO. HRV variables reflecting SNS and PNS changed dominance from SR1 to PLR, and in reverse from PLR to SR2. Thus, an initial predominant SNS in response to draining of CBV was supplanted by a relative increase in PNS (withdrawal of SNS) during PLR. This ran in parallel with the increase in SV which may be seen as a response to the augmentation of CBV.

### 4.2 Equipment and methodology

The PLR has been promulgated as a dependable and innocuous assessment of preload responsiveness in the ICU and recovery room. Its exact execution was honed in a study by Jabot showing that the sequence “semirecumbent to PLR” induces a larger increase in cardiac preload than “supine to PLR” and thus “SR to PLR” may be preferred for predicting fluid responsiveness (Jabot et al., 2009). This was later codified by Monnet and Teboul (2015). This study adhered to the Monnet & Teboul procedure to maintain comparability with the majority of studies on the subject of volume responsiveness.

Finapres was chosen as a representative of the group of minimally invasive monitors applying pulse contour analysis (PCA) incorporating the Peñáz/Wesseling Modelflow and physiocal algorithms (Geerts et al., 2011). The Finapres has been shown to agree satisfactorily with other evaluations of central hemodynamic variables (Broch et al., 2012; Rang et al., 2007; van der Spoel et al., 2012), specifically compared to thermodilution CO during baseline and PLR (Geerts et al., 2011). In a similar study, Elwan et al. (2018) used transthoracic electric bioimpedance which to the present author seems to have minor penetration in the intensive care community. Concerns have been raised after Doppler documented vasodilatation and increased flow induced by baroreflex during PLR (London et al., 1989). The Peñáz/Wesseling PCA Modelflow, however, is not based on flow measurement, but integrates the area under the arterial pressure curve from the onset to the dicrotic notch and relates this area to cardiac output through an algorithmic autocalibration. Thus, the algorithm accounts for changes in arterial tone and compliance.

The ECG was recorded for HRV analysis in the spectral aspect. This is often applied when associations between ANS activity and clinical conditions are evaluated, for example, as a prognostic index for metabolic syndrome, heart failure, and neuroprognostication (Henden et al., 2014; Honoré et al., 2019; Vistisen et al., 2014). The reflection of the ANS subdivisions in the powers of LF, HF, and the LF/HF ratio is straightforward (with reservations noted above) and generally available in digital signal analysis applying fast Fourier transformation (FFT). Malik (1996) recommended a minimum sampling frequency of 250 to 500 Hz. Indisputably, the higher the sampling frequency, the higher the resolution and the greater the chance of discovering the miniscule changes in the RR interval. Simultaneously, it is of importance whether focus is on absolute or normalized units as suggested by Malik (1996) and Malik et al. (2019). Two early studies by McHugh bears witness to this: McHugh et al. (1994, 1997). The first report dealt with a study of positional changes and spectral components in healthy volunteers and the second in postoperative CABG patients. Noteworthy is the use of absolute power of the elements of the spectral convolute and the very low sampling frequency of the electrocardiographic recording: 200 Hz. This may have contributed to the conclusion that positional changes did not induce changes in ANS responses. Furthermore, the ECG signal is prone to error and artifacts. Thus, the Kubios analysis program offers degrees of filtering. In this study, a built‐in automatic, accurate algorithm for detecting artifacts (missed, extra, and misaligned beat detections) as well as ectopic beats was applied. This feature was not used in the studies by McHugh, nor the recent publication by Sejersen et al. (2022) probably explaining the lack of significant results. Notably, the road from registration of the electrical activity of the heart to the presentations of an ECG and final results of the FFT passes several stations in digital signal analysis in the shape of electrical components, analog‐to‐digital conversion, filtering, and analytical algorithms. If this sequence creates aberrant results, they may legitimately (from a statistical point of view) be removed by application of an outlier removal algorithm. ROUT was applied in this study and may be part of the successful demonstration of differences in contradistinction to studies referred to above.

Gaffney et al. (1982) published a study noticing only a small, transient increase in stroke volume during PLR in healthy volunteers. The effect was absent after 30 to 45 min. In 1988, Wong demonstrated a 3% increase in CI in a cohort of patients. Results were reported for 5 min post‐PLR. The study was followed in 1989 by a study including a fixed volume of 500 ml hemorrhaged blood in a cohort of healthy subjects, demonstrating a slight increase in cardiac and stroke index during PLR post‐hemorrhage (Wong et al., 1989). Discussions in these three early studies of the effect of PLR centered on the gravitational dislocation of volume and the autonomic nervous reactions, although these were not actively monitored, to the maneuver. Anderson, following Wong's publication, noted that “The clinical significance of changes in cardiovascular function, after injury and hemorrhage, must ultimately lie in their influence on our ability to deliver oxygen to the tissues and our monitoring of this treatment” (Anderson, 1991). This is a tribute to Bernard's concept of the “milieu interieure” (Bernard, 1878) and Cannon's “homeostasis” (Cannon, 1939). According to Guyton, the cardiovascular system has “at least” three compensatory mechanisms (cp. homeostasis) offsetting the effects of changes in blood volume (here interpreted also as “changes in stressed volume or CBV”): nervous reflexes, stress relaxation/recovery and readjustment of blood volume by renal secretion/reabsorption and transcapillary autotransfusion/extravasation/lymphatic retransfusion via the thoracic duct (Guyton, 1963). The mechanisms have different time constants, thus reaching saturation after different time courses—reflexes acting fast, stress relaxation/recovery within minutes to hours and renal, lymphatic and transcapillary mechanisms within days. The acute events elicited by changing position from SR1 to PLR may be envisaged as an immediate increase in CBV manifesting as an increase in mean systemic filling pressure (MSFP). MSFP enters the term for venous return pressure, VRdP = MSFP‐RAP. Depending on heart efficiency, the heart may maintain a constant RAP and the increased venous return increases CO. CO increases MAP which stimulates the aortic and carotid pressoreceptors, inhibits the SNS and activates the PNS, induces vasodilation, and decreases HR. Two studies demonstrate the importance of heart efficiency in the responses to PLR: Thomas and Shillingford (1965) presented measurements of arterial pressure, CO, HR, and SV in patients with presumed normal cardiac function and patients having suffered a myocardial infarction 2–6 weeks prior to the study. Measurements were made in 20° anti‐Trendelenburg position and 3–6 min after 60° PLR. Subjects without heart disease increased SV by 35% ± 6.4%, whereas patients with a history of MI increased by 13% ± 19.9% (note: SD including instances of *decreasing* SV in response to PLR). Similarly, Si 53 years later related the PLR threshold values of ΔCO and ΔSV to Global Ejection Fraction (GEF is a transpulmonary thermodilution‐derived index of the systolic function, [Litton & Morgan, 2012]) in two groups, GEF≥20% and GEF < 20%. The high‐GEF group showed a 12% threshold of ΔSV versus 8% in the low‐GEF group and ΔCO of 7 versus 6% (Si et al., 2018). In this study, 9/11 subjects increased SV as a reflection, reasonably explained by increased VRdP and normal heart efficiency and counteracted an increase in CO by lowering MAP and HR.

#### 4.2.1 The short‐lived response to PLR

This study focused on differences in macrohemodynamics and HRV variables between positions of presumed equilibrated states, SR1, PLR, and SR2. This was borne out of the deliberation that FFT is not well suited for following rapid changes as the algorithm needs up to 5 min of registration to deliver reliable results. It is well‐known that CO immediately after change of position increases and subsequently decreases. This has led to the recommendation that only fast reacting CO measurement methods should be employed (Monnet & Teboul, 2008, 2010), even finding its way into a renowned textbook by Marik (2015). One of the first studies was performed by Gaffney et al. (1982) who studied the time course of changes in SV/CO in healthy volunteers following PLR. Measurements were performed 20 s and 7 min after taking up the 60° PLR position. CO and SV increased significantly in the 20″‐measurements but returned to baseline values within 7′. The speedy disappearance of induced changes is readily explained by reference to vascular stress relaxation as demonstrated by Prather and Guyton (1969). If a PLR‐induced endogenous volume bolus has a duration of <7 min, Hilton and Bellomo (2012) raised the question for how long does an exogenous fluid bolus affect macrohemodynamics? It was answered by Farkas (2019) and Nunes et al. (2014): neither the endo‐ nor the exogenous fluid bolus have a lasting effect in fluid responders.

Another question concerns the role of ANS in producing the concordant, immediate changes in SV and CO. Frye and Braunwald (1960) published a study with the aim of determining whether the circulatory response to acutely induced hypervolemia were modified by reducing the activity of the ANS by means of ganglionic blockade. Seven subjects underwent serial phlebotomy to a cumulated volume of 1.5 L. Within 2 days after the last phlebotomy, the volume was returned in approximately 80 min. No intervention affecting ANS was made. Hemodynamics (HR, CO, and intra‐arterial BP) were recorded before transfusion, immediately after completion and 15 min later. The phlebotomy sequence was repeated. Before transfusion of exsanguinated blood, an infusion of trimetaphan was instituted counteracting cholinergic transmission at the nicotinic receptors of the autonomic ganglia blocking the SNS and PNS. The infusion targeted a decrease in SBP of 40 mmHg. CO was determined by the indicator dilution technique. The remarkable differences were related to the absence of autonomic reflexes during ganglionic blockade, summarized in the conclusion: “Experiments in which hypervolemia is induced acutely in intact human subjects may not be ideally suited for determining the applicability of Starling's law of the heart to man. The presence of an intact autonomic nervous system results in failure of a significant fraction of the infused fluids to augment the volume of blood within the heart and lungs, and the stimulation of myocardial contraction provided by an increased end‐diastolic fiber length cannot result.” In this study, a discordance between SV and CO increases was noted: SV pointed to intact volume responsiveness, whereas CO did not. Possibly the ANS activity implied a counterregulation maintaining DO_2_ in a situation where VO_2_ was constant as no physical activity was present and by implication constant CO. In the Frye study, both SV and CO remained stable for the considerably longer period of transfusion, 80 minutes, compared to the 10 min of PLR in the present setting.

Looking further, studies identifying preload responsiveness based on PLR vary according to the chosen variable. Some favor SV (Biais et al., 2009; Preau et al., 2010; Thiel et al., 2009) while others use CO (Cherpanath et al., 2016; Monge Garcia et al., 2012; Monnet et al., 2012), both with a cut‐off of 13%–15% based on receiver operating characteristics (ROC) analysis. Two meta‐analyses demonstrated almost equal numbers of studies using CO (as aortic blood flow) and SV for the assessment of preload responsiveness. Studies using CO as the arbiter of preload responsiveness have likely been carried out in patients with attenuated autonomic and homeostatic reflexes, whereas in healthy volunteers, the differential responses of CO and SV are revealed as CO is preserved in face of unaltered “milieu intérieure.” Thus, from a clinical perspective, it is of interest to gauge the interaction between central hemodynamic variables and autonomic control during PLR in patients with septic and acute heart failure, as well as in patients exposed to sedation or anesthesia, that is, sympatholytic states. If SV and CO increases in response to PLR are associated with attenuation of the SNS index, the responses may be induced by vasoplegia, increased pooling in an unresponsive distal vascular bed during SR1, and unabated augmentation of CBV increasing MSFP in a relatively noncompliant central venous circulation during PLR. This “preload responsiveness” should be interpreted cautiously when administering external fluid and assessing the need for volume expansion rather than correction of vasodilatation.

### 4.3 Limitations

The number of volunteers was limited to 11, which was sufficient for probing the PLR and ANS. The significant results invite a larger study including both healthy persons and patients. This study addressed the relationship between HRV and PLR in healthy volunteers and directed attention to the discordant responses in SV and CO. To become clinically relevant, patients should be incorporated and the ANS should be pharmacologically manipulated. It is a limitation that a supine position was not included in the protocol for exploration of the mechanisms involved in the PLR procedure, but the study specifically addressed the recommendations provided by Monnet and Teboul (2015). From a pragmatic and clinical feasibility point of view, it is outside the scope to look for changes in CBV, for example, by regional electrical admittance (Cai et al., 2000), echocardiography (Brothers et al., 2014), and transpulmonary thermodilution for the determination of CO, intrathoracic blood volume, and global end‐diastolic volume (Reuter et al., 2010).

## 5 CONCLUSION

In conclusion, the gravitational influence on the CBV inherent to the PLR maneuver elicits a modulatory autonomic signal effecting central hemodynamic responses, preserving homeostasis of CO. In terms of SV, normal subjects appear volume responsive, whereas stable CO results from the homeostatic mechanisms of the human body.

## AUTHOR CONTRIBUTIONS

Søren Søndergaard designed the study, performed the analyses, and wrote the first and final drafts of the manuscript.

## FUNDING INFORMATION

The author declares that he did not receive external funding.

## CONFLICT OF INTEREST

The author declares that he has no competing interests.

## ETHICS APPROVAL STATEMENT

The Central Denmark Region Committees on Health Research Ethics (Chairperson Birgitte Mahler, MD) on October 4, 2017 (No. 10‐72‐212‐17).

COPE: The author fully adheres to the Committee on Publication Ethics (COPE) Code of Conduct and its Best Practice Guidelines. The report of this observational study followed the STROBE checklist of items (https://www.strobe‐statement.org).

## Data Availability

The datasets generated and/or analyzed during this study are not publicly available due to EU regulations but are available from the corresponding author upon reasonable request.

## References

[phy215537-bib-0001] Anderson, D. (1991). Passive leg raising. Critical Care Medicine, 19, 126. 10.1097/00003246-199101000-00030 1986880

[phy215537-bib-0002] Aneman, A. , & Sondergaard, S. (2016). Understanding the passive leg raising test. Intensive Care Medicine, 42, 1493–1495. 26846515 10.1007/s00134-016-4228-4

[phy215537-bib-0003] Bahlmann, H. , Hahn, R. G. , & Nilsson, L. (2016). Agreement between Pleth variability index and oesophageal doppler to predict fluid responsiveness. Acta Anaesthesiologica Scandinavica, 60, 183–192. 26373826 10.1111/aas.12632

[phy215537-bib-0004] Bernard, C. (1878). Leçons sur les phénomènes de la vie communs aux animaux et aux végétaux. J. B. Baillière et fils.

[phy215537-bib-0005] Biais, M. , Vidil, L. , Sarrabay, P. , Cottenceau, V. , Revel, P. , & Sztark, F. (2009). Changes in stroke volume induced by passive leg raising in spontaneously breathing patients: Comparison between echocardiography and Vigileo/FloTrac device. Critical Care, 13, R195. 19968880 10.1186/cc8195 PMC2811910

[phy215537-bib-0006] Bos, W. J. , Van Goudoever, J. , Van Montfrans, G. A. , Van Den Meiracker, A. H. , & Wesseling, K. H. (1996). Reconstruction of brachial artery pressure from noninvasive finger pressure measurements. Circulation, 94, 1870–1875. 8873662 10.1161/01.cir.94.8.1870

[phy215537-bib-0007] Broch, O. , Renner, J. , Gruenewald, M. , Meybohm, P. , Schottler, J. , Caliebe, A. , Steinfath, M. , Malbrain, M. , & Bein, B. (2012). A comparison of the Nexfin(R) and transcardiopulmonary thermodilution to estimate cardiac output during coronary artery surgery. Anaesthesia, 67, 377–383. 22324797 10.1111/j.1365-2044.2011.07018.x

[phy215537-bib-0008] Brothers, R. M. , Pecini, R. , Dalsgaard, M. , Bundgaard‐Nielsen, M. , Wilson, T. E. , Secher, N. H. , & Crandall, C. G. (2014). Beneficial effects of elevating cardiac preload on left‐ventricular diastolic function and volume during heat stress: Implications toward tolerance during a hemorrhagic insult. American Journal of Physiology. Regulatory, Integrative and Comparative Physiology, 307, R1036–R1041. 25163916 10.1152/ajpregu.00151.2014 PMC4200382

[phy215537-bib-0009] Cai, Y. , Holm, S. , Jenstrup, M. , Stromstad, M. , Eigtved, A. , Warberg, J. , Hojgaard, L. , Friberg, L. , & Secher, N. H. (2000). Electrical admittance for filling of the heart during lower body negative pressure in humans. Journal of Applied Physiology (1985), 89, 1569–1576. 10.1152/jappl.2000.89.4.1569 11007597

[phy215537-bib-0010] Cannon, W. B. (1939). The wisdom of the body. W.W. Norton & Company.

[phy215537-bib-0011] Cherpanath, T. G. , Hirsch, A. , Geerts, B. F. , Lagrand, W. K. , Leeflang, M. M. , Schultz, M. J. , & Groeneveld, A. B. (2016). Predicting fluid responsiveness by passive leg raising: A systematic review and meta‐analysis of 23 clinical trials. Critical Care Medicine, 44, 981–991. 26741579 10.1097/CCM.0000000000001556

[phy215537-bib-0012] De Backer, D. , Cecconi, M. , Lipman, J. , Machado, F. , Myatra, S. N. , Ostermann, M. , Perner, A. , Teboul, J. L. , Vincent, J. L. , & Walley, K. R. (2019). Challenges in the management of septic shock: A narrative review. Intensive Care Medicine, 45, 420–433. 30741328 10.1007/s00134-019-05544-x

[phy215537-bib-0013] Elwan, M. H. , Roshdy, A. , Reynolds, J. A. , Elsharkawy, E. M. , Eltahan, S. M. , & Coats, T. J. (2018). What is the normal haemodynamic response to passive leg raise? A study of healthy volunteers. Emergency Medicine Journal, 35, 544–549. 29728410 10.1136/emermed-2017-206836

[phy215537-bib-0014] Farkas, J. (2019). Myth‐busting the fluid bolus . [Online]. Retrieved September 9, 2022, from https://emcrit.org/pulmcrit/bolus/

[phy215537-bib-0015] Frye, R. L. , & Braunwald, E. (1960). Studies on Starling's law of the heart. I. The circulatory response to acute hypervolemia and its modification by ganglionic blockade. The Journal of Clinical Investigation, 39, 1043–1050. 13825478 10.1172/JCI104119 PMC441848

[phy215537-bib-0016] Gaffney, F. A. , Bastian, B. C. , Thal, E. R. , Atkins, J. M. , & Blomqvist, C. G. (1982). Passive leg raising does not produce a significant or sustained autotransfusion effect. The Journal of Trauma, 22, 190–193. 7069801 10.1097/00005373-198203000-00003

[phy215537-bib-0017] Geerts, B. , De Wilde, R. , Aarts, L. , & Jansen, J. (2011). Pulse contour analysis to assess hemodynamic response to passive leg raising. Journal of Cardiothoracic and Vascular Anesthesia, 25, 48–52. 21093293 10.1053/j.jvca.2010.09.013

[phy215537-bib-0018] Guelen, I. , Westerhof, B. E. , Van Der Sar, G. L. , Van Montfrans, G. A. , Kiemeneij, F. , Wesseling, K. H. , & Bos, W. J. (2003). Finometer, finger pressure measurements with the possibility to reconstruct brachial pressure. Blood Pressure Monitoring, 8, 27–30. 12604933 10.1097/00126097-200302000-00006

[phy215537-bib-0019] Guyton, A. C. (1963). Cardiac output and its regulation. Saunders.

[phy215537-bib-0020] Henden, P. L. , Sondergaard, S. , Rydenhag, B. , Reinsfelt, B. , Ricksten, S. E. , & Aneman, A. (2014). Can baroreflex sensitivity and heart rate variability predict late neurological outcome in patients with traumatic brain injury? Journal of Neurosurgical Anesthesiology, 26, 50–59. 24064714 10.1097/ANA.0b013e3182a47b62

[phy215537-bib-0021] Hilton, A. K. , & Bellomo, R. (2012). A critique of fluid bolus resuscitation in severe sepsis. Critical Care, 16, 302. 22277834 10.1186/cc11154 PMC3396245

[phy215537-bib-0022] Honoré, H. , Eggertsen, K. , & Sondergaard, S. (2019). A study into the feasibility of using HRV variables to guide treatment in patients with paroxystic sympathetic hyperactivity in a neurointensive step‐down unit. NeuroRehabilitation, 44, 141–155. 30741702 10.3233/NRE-182557

[phy215537-bib-0023] Iwase, S. , Hayano, J. , & Orimo, S. (2017). Clinical assessment of the autonomic nervous system. Springer.

[phy215537-bib-0057] Jabot, J. , Teboul, J. L. , Richard, C. , & Monnet, X. (2009). Passive leg raising for predicting fluid responsiveness: Importance of the postural change. Intensive Care Medicine, 35, 85–90. 18795254 10.1007/s00134-008-1293-3

[phy215537-bib-0024] Litton, E. , & Morgan, M. (2012). The PiCCO monitor: A review. Anaesthesia and Intensive Care, 40, 393–409. 22577904 10.1177/0310057X1204000304

[phy215537-bib-0025] London, G. M. , Pannier, B. M. , Laurent, S. , & Safar, M. E. (1989). Cardiopulmonary baroreflex control of brachial artery diameter in sustained essential hypertension. Journal of Hypertension, 7, 879–883. 2607141 10.1097/00004872-198911000-00005

[phy215537-bib-0026] Malik, M. (1996). Heart rate variability. Standards of measurement, physiological interpretation, and clinical use. Task force of the European Society of Cardiology and the north American Society of Pacing and Electrophysiology. European Heart Journal, 17, 354–381. 8737210

[phy215537-bib-0027] Malik, M. , Hnatkova, K. , Huikuri, H. V. , Lombardi, F. , Schmidt, G. , & Zabel, M. (2019). CrossTalk proposal: Heart rate variability is a valid measure of cardiac autonomic responsiveness. The Journal of Physiology, 597, 2595–2598. 31006862 10.1113/JP277500 PMC6826215

[phy215537-bib-0028] Marik, P. E. (2015). Evidence‐based critical care. Springer.

[phy215537-bib-0029] Mchugh, G. J. , Robinson, B. J. , & Galletly, D. C. (1994). Leg elevation compared with Trendelenburg position: Effects on autonomic cardiac control. British Journal of Anaesthesia, 73, 836–837. 7880676 10.1093/bja/73.6.836

[phy215537-bib-0030] Mchugh, G. J. , Sleigh, J. W. , Bo, H. , & Henderson, J. D. (1997). Heart rate variability following cardiac surgery fails to predict short‐term cardiovascular instability. Anaesthesia and Intensive Care, 25, 621–626. 9452842 10.1177/0310057X9702500604

[phy215537-bib-0031] Michard, F. , & Teboul, J. L. (2002). Predicting fluid responsiveness in ICU patients: A critical analysis of the evidence. Chest, 121, 2000–2008. 12065368 10.1378/chest.121.6.2000

[phy215537-bib-0032] Monge Garcia, M. I. , Gil Cano, A. , Gracia Romero, M. , Monterroso Pintado, R. , Perez Madueno, V. , & Diaz Monrove, J. C. (2012). Non‐invasive assessment of fluid responsiveness by changes in partial end‐tidal CO_2_ pressure during a passive leg‐raising maneuver. Annals of Intensive Care, 2, 9. 22449292 10.1186/2110-5820-2-9 PMC3327636

[phy215537-bib-0033] Monnet, X. , Bleibtreu, A. , Ferre, A. , Dres, M. , Gharbi, R. , Richard, C. , & Teboul, J. L. (2012). Passive leg‐raising and end‐expiratory occlusion tests perform better than pulse pressure variation in patients with low respiratory system compliance. Critical Care Medicine, 40, 152–157. 21926581 10.1097/CCM.0b013e31822f08d7

[phy215537-bib-0034] Monnet, X. , & Teboul, J. L. (2008). Passive leg raising. Intensive Care Medicine, 34, 659–663. 18214429 10.1007/s00134-008-0994-y

[phy215537-bib-0035] Monnet, X. , & Teboul, J. L. (2010). Passive leg raising: Keep it easy! Intensive Care Medicine, 36, 1445 author reply 446. 20495783 10.1007/s00134-010-1900-y

[phy215537-bib-0036] Monnet, X. , & Teboul, J. L. (2015). Passive leg raising: Five rules, not a drop of fluid! Critical Care, 19, 18. 25658678 10.1186/s13054-014-0708-5 PMC4293822

[phy215537-bib-0037] Motulsky, H. J. , & Brown, R. E. (2006). Detecting outliers when fitting data with nonlinear regression—A new method based on robust nonlinear regression and the false discovery rate. BMC Bioinformatics, 7, 123. 16526949 10.1186/1471-2105-7-123 PMC1472692

[phy215537-bib-0038] Nunes, T. S. , Ladeira, R. T. , Bafi, A. T. , De Azevedo, L. C. , Machado, F. R. , & Freitas, F. G. (2014). Duration of hemodynamic effects of crystalloids in patients with circulatory shock after initial resuscitation. Annals of Intensive Care, 4, 25. 25593742 10.1186/s13613-014-0025-9 PMC4273721

[phy215537-bib-0039] Peñáz, J. , Voigt, A. , & Teichmann, W. (1976). Beitrag zur fortlaufender indirekten Blutdruckmessung. Zeitschrift für die gesamte innere Medizin und ihre Grenzgebiete, 31, 1030–1033. 1020409

[phy215537-bib-0040] Prather, J. W. , Taylor, A. E. , & Guyton, A. C. (1969). Effect of blood volume, mean circulatory pressure, and stress relaxation on cardiac output. The American Journal of Physiology, 216, 467–472. 5765598 10.1152/ajplegacy.1969.216.3.467

[phy215537-bib-0041] Preau, S. , Saulnier, F. , Dewavrin, F. , Durocher, A. , & Chagnon, J. L. (2010). Passive leg raising is predictive of fluid responsiveness in spontaneously breathing patients with severe sepsis or acute pancreatitis. Critical Care Medicine, 38, 819–825. 20016380 10.1097/CCM.0b013e3181c8fe7a

[phy215537-bib-0042] Rang, S. , De Pablo Lapiedra, B. , Van Montfrans, G. A. , Bouma, B. J. , Wesseling, K. H. , & Wolf, H. (2007). Modelflow: A new method for noninvasive assessment of cardiac output in pregnant women. American Journal of Obstetrics and Gynecology, 196(235), e1–e8. 10.1016/j.ajog.2006.10.896 17346534

[phy215537-bib-0043] Reuter, D. A. , Huang, C. , Edrich, T. , Shernan, S. K. , & Eltzschig, H. K. (2010). Cardiac output monitoring using indicator‐dilution techniques: Basics, limits, and perspectives. Anesthesia and Analgesia, 110, 799–811. 20185659 10.1213/ANE.0b013e3181cc885a

[phy215537-bib-0044] Sejersen, C. , Christiansen, T. , & Secher, N. H. (2022). To identify normovolemia in humans: The stroke volume response to passive leg raising vs. head‐down tilt. Physiological Reports, 10, e15216. 35854636 10.14814/phy2.15216 PMC9296869

[phy215537-bib-0045] Si, X. , Cao, D. Y. , Chen, J. , Wu, J. F. , Liu, Z. M. , Xu, H. L. , Chen, M. Y. , Liu, Y. J. , & Guan, X. D. (2018). Effect of systolic cardiac function on passive leg raising for predicting fluid responsiveness: A prospective observational study. Chinese Medical Journal, 131, 253–261. 29363638 10.4103/0366-6999.223841 PMC5798044

[phy215537-bib-0046] Sondergaard, S. (2013). Pavane for a pulse pressure variation defunct. Critical Care, 17, 327. 24229428 10.1186/cc13109 PMC4056112

[phy215537-bib-0047] Stick, S. (1996). Measurements during tidal breathing. In J. Stocks , P. D. Sly , R. S. Tepper , & J. W. Morgan (Eds.), Infant respiratory function testing. Wiley‐Liss.

[phy215537-bib-0048] Sullivan, L. M. , Weinberg, J. , & Keaney, J. F., Jr. (2016). Common statistical pitfalls in basic science research. Journal of the American Heart Association, 5, 1–9. 10.1161/JAHA.116.004142 PMC5121512 27688237

[phy215537-bib-0049] Tarvainen, M. , Lipponen, J. , Niskanen, J.‐P., Jr. , & Ranta‐Aho, P. (2021). Kubios HRV (ver. 3.5), USER'S GUIDE HRV Standard HRV Premium.

[phy215537-bib-0050] Thiel, S. W. , Kollef, M. H. , & Isakow, W. (2009). Non‐invasive stroke volume measurement and passive leg raising predict volume responsiveness in medical ICU patients: An observational cohort study. Critical Care, 13, R111. 19586543 10.1186/cc7955 PMC2750155

[phy215537-bib-0051] Thomas, M. , & Shillingford, J. (1965). The circulatory response to a standard postural change in Ischaemic heart disease. British Heart Journal, 27, 17–27. 14242159 10.1136/hrt.27.1.17 PMC490130

[phy215537-bib-0052] Van Der Spoel, A. G. , Voogel, A. J. , Folkers, A. , Boer, C. , & Bouwman, R. A. (2012). Comparison of noninvasive continuous arterial waveform analysis (Nexfin) with transthoracic doppler echocardiography for monitoring of cardiac output. Journal of Clinical Anesthesia, 24, 304–309. 22608585 10.1016/j.jclinane.2011.09.008

[phy215537-bib-0053] Vistisen, S. T. , Hansen, T. K. , Jensen, J. , Nielsen, J. F. , & Fleischer, J. (2014). Heart rate variability in neurorehabilitation patients with severe acquired brain injury. Brain Injury, 28, 196–202. 24295072 10.3109/02699052.2013.860477

[phy215537-bib-0054] Wesseling, K. H. , Jansen, J. R. , Settels, J. J. , & Schreuder, J. J. (1993). Computation of aortic flow from pressure in humans using a nonlinear, three‐element model. Journal of Applied Physiology, 74, 2566–2573. 8335593 10.1152/jappl.1993.74.5.2566

[phy215537-bib-0055] Wong, D. H. , O'connor, D. , Tremper, K. K. , Zaccari, J. , Thompson, P. , & Hill, D. (1989). Changes in cardiac output after acute blood loss and position change in man. Critical Care Medicine, 17, 979–983. 2791582 10.1097/00003246-198910000-00002

[phy215537-bib-0056] Zhang, Y. , Hedo, R. , Rivera, A. , Rull, R. , Richardson, S. , & Tu, X. M. (2019). Post hoc power analysis: Is it an informative and meaningful analysis? General Psychiatry, 32, e100069. 31552383 10.1136/gpsych-2019-100069 PMC6738696

